# Editorial: Tendons and ligaments: development, pathogenesis, tissue engineering, and regenerative medicine

**DOI:** 10.3389/fbioe.2024.1489256

**Published:** 2024-09-17

**Authors:** Dai Fei Elmer Ker, Cheemeng Tan, Sarah Cartmell

**Affiliations:** ^1^ Department of Biomedical Engineering, The Hong Kong Polytechnic University, Hung Hom, Hong Kong SAR, China; ^2^ Department of Biomedical Engineering, University of California, Davis, CA, United States; ^3^ Department of Materials Science, Henry Royce Institute, School of Natural Sciences, Faculty of Science and Engineering, The University of Manchester, Manchester, United Kingdom

**Keywords:** tendon, ligament, tissue engineering, regenerative medicine, animal model, stem cell, growth factors

Globally, musculoskeletal diseases and injuries, such as tendon and ligament deficits, drastically reduce patient quality-of-life and increase national healthcare costs (WHO Scientific Group on the Burden of Musculoskeletal Conditions at the Start of the New Millennium, 2003; United States Bone and Joint Initiative, 2014; Global Burden of Disease, 2016 Disease and Injury Incidence and Prevalence Collaborators, 2017). In developed regions, such as the United States (Mather and Scommegna, 2024), United Kingdom (Centre for Aging Better, 2023), and Hong Kong (Census and Statistics Department of Hong Kong, 2015), this undesirable effect is exacerbated by rapid population aging. Addressing this challenge requires key advances to bridge both knowledge and technical gaps in musculoskeletal science, tissue engineering, and regenerative medicine. In this regard, the “*Tendons and Ligaments: Development, Pathogenesis, Tissue Engineering, and Regenerative Medicine*” Research Topic highlights some of the latest unique and groundbreaking preclinical and clinical studies.

This Research Topic features concise reviews and innovative research articles that summarize and/or showcase the latest advances in tendon/ligament basic and translational science.

The concise reviews herein present an overview of the tendon wound healing environment (Hart et al.) as well as pro-regenerative factors such as growth factors (Lin et al.), non-coding RNAs (Silva et al.), and tendon stem/progenitor cells (He et al.). Hart et al. discuss the importance of the Achilles tendon wound environment following injury along with surgical and non-surgical treatment options, state-of-the-art therapeutics under development, and the necessity of biomarkers that can be prognostic of long-term tendon healing outcomes to improve clinical research and clinical trials. Lin et al. summarize recent advances and limitations in growth factor-based tendon regenerative strategies, which include the combinatorial use of stem cells and scaffolds. The latter also highlights the need for more studies to support their use for routine management of tendon ailments. As an alternative to growth factors, Silva et al. describe the use of non-coding RNAs, such as siRNAs, miRNAs, and lncRNAs, as molecular tools for tendon tissue engineering. These efforts aim to knockdown proteins associated with detrimental biological processes such as fibrosis and excessive inflammation or to beneficially enhance tendon healing. Providing an added dimension to these topics, He et al. examine cell-based strategies such as tendon-derived stem cells for creating tissue-engineered constructs, including the use of pro-regenerative cues, bioengineered cell sheets biomaterial scaffolds, and bioreactor-based mechanical conditioning.

Original research articles within this Research Topic include publications that advance the frontiers of scientific and clinical knowledge. These publications highlight trending tendon/ligament Research Topics (Zhang et al.), showcase current clinical opinions and practices (Xue et al.), elucidate the contribution of ECM in tendon/ligament biomechanics (Liu et al.), improve diagnosis of tendon pulley injuries (Iruretagoiena et al.), characterize novel drug delivery platforms (Shi et al.), and bioengineer stem cell-based tendon constructs (Taguchi et al.). Serving as a reference for the tendon/ligament field, Zhang et al. conducted bibliometric analyses to uncover researchers and the regions where tendon/ligament research is being actively conducted together with trending researching topics such as biomaterial scaffolds and immunomodulatory strategies. Meanwhile, Xue et al. conducted a survey among British hand surgeons to better understand the current treatments preferred by medical practitioners as well as their opinion on employing state-of-the-art tools and techniques such as minimally invasive instruments, biodegradable materials, and additive manufactured devices. The results reflect a conservative approach among clinicians with a weak preference for new techniques and advances. Altogether, such information is crucial for assessing the current state and future progress in tendon/ligament science and clinical practice. Liu et al. studied the contribution of elastin to patellar tendon biomechanics by subjecting porcine patellar tendons to elastin digestion followed by macroscopic mechanical testing. The study demonstrated that elastin plays an important role in the mechanical properties and fiber structure stability of patellar tendon, contributing towards our understanding of the structure-function relationship of patellar tendon. Iruretagoiena et al. performed high-resolution ultrasound measurements of tendon-to-bone distances. This work enabled the authors to uncover a minimum threshold for tendon-to-bone distance to diagnose partial and complete flexor tendon pulley injuries, which are common in rock climbers. Shi et al. characterized osteoadsorptive fluorogenic sentinel 3 (OFS-3), a novel drug delivery platform that comprises a bone-targeting bisphosphonate (BP) and cathepsin K (Ctsk)-triggered compound. In this study, the authors ruled out any potential negative effects on tendon biomechanical attributes and tendon healing efficacy, which paves the way for this drug delivery platform to undergo further development to specifically deliver therapeutic agents such as growth factors to bone-tendon sites. In Taguchi et al., the authors generated tendon tissue engineered constructs using a combination of adipose-derived stem cells. Collagen Type I sponge, tenogenic media, and tensile bioreactor conditioning. This work shows promise for contributing to the development of novel tendon therapies.

Collectively, the diverse works contained within the “*Tendons and Ligaments: Development, Pathogenesis, Tissue Engineering, and Regenerative Medicine*” Research Topic illustrate the latest conceptual and technical advances for tendon/ligament basic and clinical sciences. These publications highlight current challenges in tendon/ligament regeneration, describe recent scientific progress, and present potential solutions, offering a promising outlook for this field.
